# The Pacific Medical Supply Workers Buddy Network: a regional professionalization activity

**DOI:** 10.1186/2052-3211-7-S1-O10

**Published:** 2014-12-17

**Authors:** Ben Gilbert, Andrew Brown

**Affiliations:** 1 University of Canberra, Canberra, Australia; 2 People that Deliver, Copenhagen, Denmark

## Background

Field experience, literature review and focus group findings indicated that a Buddy Network could help to overcome the unique barriers encountered by senior medical supply workers in Pacific Island Countries, including chief pharmacists, central medical store managers and country program managers. Specifically it could: encourage shared problem solving, reduce professional isolation, provide a proxy of supervision and encourage self-direction, improve workplace confidence minimise training fatigue, and maximise application of training.

## Method

Utilising the researchers existing relationships, senior medical supply workers in Pacific Island Countries were invited to join the Pacific Medical Supply Workers Buddy Network. Further invitations were sent as members identified suitable colleagues to join. Members committed to sharing work achievements and challenges with each other via email, the Network website http://pacificmed.net/ and a monthly newsletter. Quantitative and qualitative evaluation of satisfaction with, and performance of, the Network was undertaken at 6 and 12 months.

## Results

Eleven newsletters have been published, based on approximately 2000 communication episodes. On a 7 point scale (1=not at all, 4=neutral, 7=very much) 9 of 23 members report that their initial hopes of the Network have been met “a lot” (5.9/7).

In descending order of positive response, members consider that the Buddy Network:

1. reduces professional isolation (6.2/7) (a lot)

2. improves workplace confidence (5.7/7) (a lot)

3. maximises application of training (5.3/7) (a little)

4. encourages shared problem solving (5.2/7) (a little)

5. provides a proxy of supervision and encourages self-direction (5.2/7) (a little)

6. minimises training fatigue (4.2/7) (neutral)

## Discussion

The Network was established to help senior medical supply workers “learn from each other’s challenges and experiences – approaching our situation with new ideas”. Current membership includes 34 senior workers from 15 countries, representing medical supply systems serving more than 9 million people. The Network has shown positive influences on many aspects of member’s work, with the expectation that this will improve their ability to manage their countries medical supply systems and thereby improve access to medical supplies in their countries. Members consider unequal member contribution and difficult access to the website as the main areas requiring improvement in the Network.

## Lessons learned

• Email is the preferred method of communication.

• The potential website benefits (resource library, chat, discussion forum) have not yet been realised due to restricted or expensive internet access and some unfamiliarity with technology.

• Active members expect greater contributions by less active members and look to the researchers to drive this.

